# Association of high sensitivity C-reactive protein with tearing of the long head of the biceps tendon

**DOI:** 10.1186/s12891-019-2908-2

**Published:** 2019-11-07

**Authors:** Ji-Yong Gwark, Hyung Bin Park

**Affiliations:** Department of Orthopaedic Surgery, Gyeongsang National University School of Medicine, Gyeongsang National University Changwon Hospital, 11 Samjeongja-ro Seongsan-gu, Changwon, 51472 Republic of Korea

**Keywords:** Biceps tendon tears, Hs-CRP, Pain, Rotator cuff tear, Risk factor

## Abstract

**Background:**

This study proposed to investigate whether high-sensitivity C-reactive protein (hs-CRP) is an independent risk factor for long head of biceps tendon (LHBT) tear and whether hs-CRP can increase accuracy in diagnosing LHBT tear.

**Methods:**

This study involved 582 shoulders of 557 consecutive patients who received arthroscopic examinations at the authors’ institution between January 2010 and July 2018. The strengths of associations between LHBT tear and various factors were determined by calculating the odds ratios (ORs), with 95% confidence intervals (CIs), using logistic regression analyses. The studied variables were demographic, physical, social, metabolic, comorbidity, hs-CRP, and pain on a visual analog scale (VAS) factors, as well as those related to rotator cuff tear (RCT). Significant factors in the multivariable logistic analysis were evaluated to determine their diagnostic values, including their likelihood ratios and post-test probabilities for LHBT tear.

**Results:**

In the multivariable analysis, five variables were significant: age, retraction degree of Patte, subscapularis tendon tear, hs-CRP > 1 mg/L, and pain VAS (*p* ≤ 0.01). The best combination of determinations for diagnosing LHBT tear, which yielded a strong positive likelihood ratio of 19.07 and a high post-test probability of 96%, was age ≥ 67 years, subscapularis tendon tear, grade of Patte ≥2, hs-CRP > 1, and pain VAS ≥ 7.

**Conclusions:**

Serum hs-CRP > 1 mg/L is an independent risk factor for LHBT tear, along with the expected risk factors of age, subscapularis tendon tear, retraction degree of Patte, and pain VAS. Serum hs-CRP > 1 mg/L increases the diagnostic accuracy for LHBT tear.

**Level of evidence:**

Level IV, Clinical case series.

## Background

Tears of the long head of the biceps tendon (LHBT) are a significant source of anterior shoulder pain [1, 2]. The prevalence of LHBT lesions observed during arthroscopic examinations reportedly ranges from 5 to 63% [3–5]. Isolated LHBT tears are uncommon; most tears are secondary lesions associated with other shoulder pathologies [6].

Risk factors of biceps tendinitis were age [7] and other structural factors including spur on the bicipital groove [8], superior labral anterior posterior tears [9], narrowed coracohumeral distance [10], subscapularis tendon tear [8, 11, 12], and chronicity of RCT [11, 13]. Among shoulder pathologies, rotator cuff tear (RCT) is the most frequently reported as having an association with LHBT tear [3, 14, 15]. In spite of the identification of LHBT tear as a pain source in chronic massive or irreparable RCT and of the use of biceps tenotomy in reducing that pain, the pathophysiology of the induction of pain is not completely understood. Because of the strong association between the sympathetic nervous system and various pain syndromes, that shoulder pain is thought to come from sympathetic innervation in the LHBT [16, 17]. It has been observed that the pain of patients with chronic RCT is reduced after the spontaneous development of LHBT rupture [18, 19]. Inflammation is a common cause of pain, and a recent study suggests that inflammation in the rotator cuff interval signaled by rupture of the long head of the biceps is a harbinger of rotator cuff disease [11]. Although the exact causes of biceps tendinopathy are unknown, intrinsic degeneration and inflammation are accepted processes of tendinopathy of LHBT [2, 20]. However, few studies have verified whether inflammation is associated with LHBT tear. Recently, Schmalzl et al. [21] reported that the gene expression of the proinflammatory cytokines of IL-1A, IL-1B, and TNF-α and of the catabolic enzymes MMP-1, − 3, − 9, and − 13 was upregulated, whereas the expression of the anti-inflammatory gene TIMP1 was downregulated in biceps tendinitis patients. The difference between this study and those previous studies is that this study newly focused on the inflammatory biomarker, hs-CRP, as an independent risk factor.

C-reactive protein (CRP) has been widely used as a marker of inflammation for clinical practice. The standard CRP measure for monitoring inflammation is CRP > 5–10 mg/L; however, the high-sensitivity CRP (hs-CRP) measurement has been developed to increase the sensitivity in detecting CRP < 1 mg/L [22]. In particular, hs-CRP is widely accepted as a potential risk predictor for several chronic inflammatory diseases, including atherosclerosis and cardiovascular disease [23]. Increasingly, hs-CRP is becoming a useful tool in identifying low-grade chronic inflammation [24]. Previously, there was a report that the increase in CRP was correlated with the severity of upper-extremity overuse disorders [25] and frozen shoulder [26]. In other musculoskeletal research, elevated levels of hs-CRP have been reported to be associated with osteoarthritis [27, 28], chronic low back pain [29], and sciatic pain [29].

This study proposed to investigate whether high-sensitivity C-reactive protein (hs-CRP) is an independent risk factor for long head of biceps tendon (LHBT) tear and whether hs-CRP can increase accuracy in diagnosing LHBT tear.

## Methods

From January 2010 to July 2018, the 627 patients who received an arthroscopic examination performed at author’s institution by the senior author (H.B.P.) were enrolled in this study. All data on the variables related to the RCT and the arthroscopic findings were prospectively collected. (Arthrex, Naples, FL, USA).

**Fig. 1:**
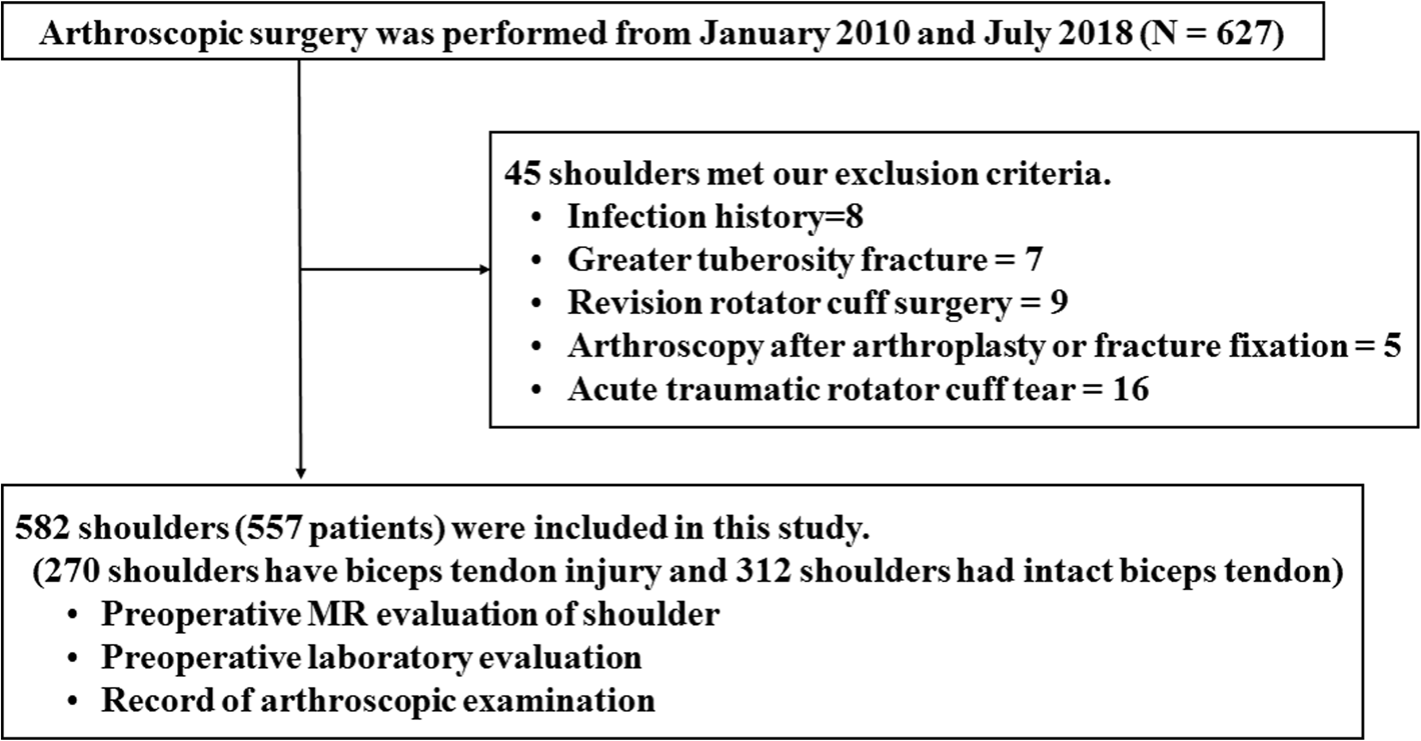
Flowchart for exclusion and inclusion criteria for this study

Of those 627 patients, 45 patients were excluded from this study for the following reasons: infection (8 patients), greater tuberosity fractures (7 patients), revision rotator cuff surgery (9 patients), arthroscopy after arthroplasty or fracture fixation (5 patients), and acute traumatic RCT (16 patients). Included in this study were 582 shoulders of the remaining 557 patients (301 males and 256 females) (Fig. 1). Of these shoulders, 345 (59.3%) were right shoulders, and 237 (40.7%) were left shoulders. The mean age at the time of arthroscopic surgery was 60.9 ± 13.2 years. The shoulder problems were classified as follows: RCT (349), impingement lesion without RCT (17), SLAP lesion excluding type 1 (80), glenohumeral instability (79), isolated LHBT lesion (10), frozen shoulder (24), and calcific tendinitis (19). Those included the following 27 cases of combined lesions: 9 cases of RCT and SLAP, 2 cases of RCT and glenohumeral instability, 8 cases of SLAP and glenohumeral instability, 3 cases of SLAP and impingement without RCT, and 5 cases of SLAP and frozen shoulder.

The studied demographic factors were age and gender. The physical factors were body mass index (BMI) and dominant-side involvement. The social factor was smoking. The medical comorbidities were diabetes, hypertension, hyperthyroidism, hypothyroidism, and the American Society of Anesthesiologists (ASA) grade. The metabolic factors were serum lipid profiles. The factors related to RCT were its presence, thickness, size, and chronicity. The chronicity indexes of RCT included the retraction degree of Patte [30], the Goutallier’s grade [31], the global fatty degeneration index (GFDI) [32], the tangent sign [33], and the occupation ratio [34]. Serum hs-CRP was measured to detect inflammation, using the AU 5800 analytical system (Beckman Coulter, CA, USA); an abnormal value was considered to be above 1 mg/L. The blood samplings were collected through venipuncture after 8 h fasting. Patients’ complaints of pain were measured using the pain VAS.

Regarding tear thickness, rotator cuff tendons were classified as intact, partial-thickness tear, or full-thickness tear. The sizes of full-thickness tears were classified as small (< 1 cm), medium (1 to 3 cm), large (3 to 5 cm), or massive (5 cm), according to the arthroscopic findings [35]. The retraction degrees of Patte were divided into three stages: in stage 1, the proximal tendon stump was close to the bony insertion; in stage 2, the proximal stump reached the level of the humeral head; and in stage 3, the proximal stump was at the level of the glenoid [30]. The Goutallier’s grade, infraspinatus muscle fatty degeneration, was determined as follows: stage 0 indicated completely normal muscle without any fatty streaks; stage 1 indicated muscle with some fatty streaks; stage 2 indicated less fat than muscle; stage 3 indicated equal amounts of fat and muscle; and stage 4 indicated a higher amount of fat than muscle [31]. The GFDI was calculated as the mean value of the grades for the supraspinatus, infraspinatus, and subscapularis muscles’ atrophy [32]. The tangent sign was evaluated, using T1-weighted oblique-sagittal images. Based on a tangent line drawn through the superior margin of the scapular spine and the superior margin of the coracoid process, the tangent sign was graded as negative if the supraspinatus muscle crossed the tangent line or positive if the supraspinatus muscle did not cross the tangent line [33]. The occupation ratio was measured, using T1-weighted oblique-sagittal images at the medial margin of coracoid process. The ratio of the area of the supraspinatus muscle to the entire supraspinatus fossa yielded the degree of atrophy of the supraspinatus muscle [34]. Table 1 summarizes demographic data on specific factors related to RCTs.

**Table 1:** Data on investigated variables in study groups

Studied variables	Biceps tendon tear (*n* = 270)	Intact biceps tendon (*n* = 312)
Age (yr)	64.9 ± 10.6	57.3 ± 14.2
< 30	1 (0.4%)	20 (6.4%)
30 to 39	9 (3.3%)	17 (5.4%)
40 to 49	8 (3.0%)	33 (10.6%)
50 to 59	55 (20.4%)	85 (27.2%)
60 to 69	85 (31.5%)	102 (32.7%)
≥ 70	112 (41.5%)	55 (17.6%)
Male gender	158 (58.5%)	160 (51.3%)
BMI (kg/m^2^)	24.1 ± 2.9	24. 4 ± 3.3
Dominant side-involvement	191 (70.7%)	212 (67.9%)
Smoking	100 (37.0%)	107 (34.3%)
Diabetes	47 (17.4%)	45 (14.4%)
Hypertension	95 (35.2%)	108 (34.6%)
Hyperthyroidism	15 (5.6%)	15 (4.8%)
Hypothyroidism	9 (3.3%)	4 (1.3%)
ASA grade		
Normal health	30 (11.1%)	56 (17.9%)
Mild systemic disease	163 (60.4%)	166 (53.2%)
Severe systemic disease	77 (28.5%)	90 (28.8%)
Prevalence of dyslipidemia	130 (48.1%)	298 (95.5%)
Hyper-cholesterolemia	62 (23.0%)	77 (24.7%)
Hyper-TGmia	64 (23.7%)	83 (26.6%)
Hyper-LDLemia	184 (68.1%)	228 (73.1%)
Hypo-HDLemia	88 (32.6%)	111 (35.6%)
Hyper-non-HDLemia	156 (57.8%)	191 (61.2%)
Presence of rotator cuff tear	183 (67.8%)	166 (53.2%)
Supraspinatus tendon tear	152 (56.3%)	136 (43.6%)
Infraspinatus tendon tear	77 (28.5%)	41 (13.1%)
Subscapularis tendon tear	110 (40.7%)	48 (15.4%)
Depth of rotator cuff tears		
Partial-thickness	39 (14.4%)	71 (22.8%)
Full-thickness	144 (53.3%)	95 (30.4%)
Size of rotator cuff tear		
Small	6 (2.2%)	16 (5.1%)
Medium	60 (22.2%)	47 (15.1%)
Large	52 (19.3%)	24 (7.7%)
Massive	26 (9.6%)	8 (2.6%)
Retraction degree of Patte		
Stage I	29 (10.7%)	41 (13.1%)
Stage II	77 (28.5%)	49 (15.7%)
Stage III	38 (14.1%)	5 (1.6%)
Goutallier’s fatty degeneration grade		
Stage I	97 (35.9%)	201 (64.4%)
Stage II	89 (33.0%)	86 (27.6%)
Stage III	59 (21.9%)	40 (12.8%)
Stage IV	14 (5.2%)	6 (1.9%)
Global fatty degeneration index	0.7 (0.3 to 1.7)	0.6 (0.2 to 1.2)
Positive tangent sign	113 (41.9%)	82 (26.3%)
Occupation ratio		
Grade I	161 (59.6%)	247 (79.2%)
Grade II	71 (26.3%)	42 (13.5%)
Grade III	38 (14.1%)	23 (7.4%)
hs-CRP (mg/L)	0.6 (0.4 to 1.5)	0.5 (0.4 to 1.2)
Pain VAS	7.5 (6.0 to 8.0)	6.0 (6.0 to 7.0))

* means that significant values in univariate logistic regression analysis

**Fig. 2:**
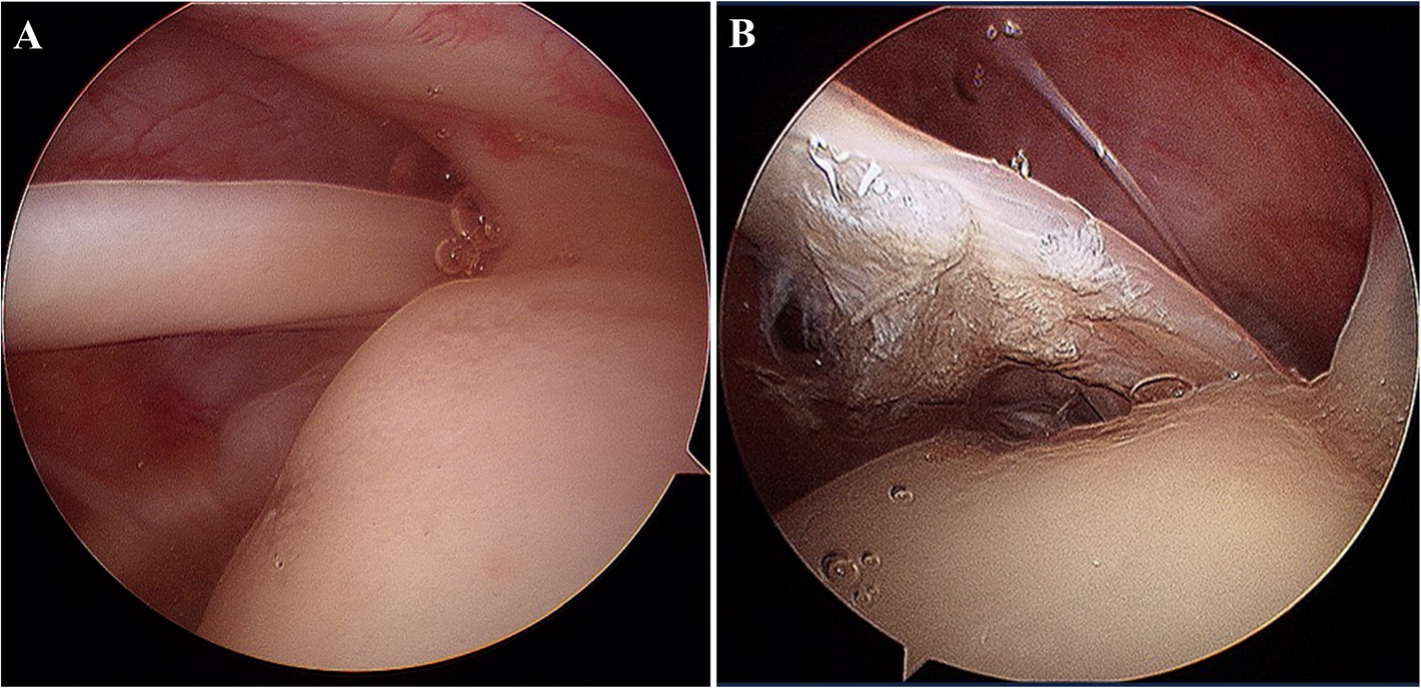
Arthroscopic findings regarding the integrity of the long head of biceps tendons. **a** The long head of a biceps tendon classified as intact, using arthroscopic findings. **b** The long head of a biceps tendon classified as a tear, using arthroscopic findings

The diagnosis of LHBT tear was made according to the Laffose et al. [14] classification, based on arthroscopic findings. Each LHBT was assigned to grade 0 (normal tendon), or to grade I (minor lesion with fraying or erosion involving < 50% of the tendon diameter), or to grade II (major lesion with fraying or erosion involving > 50% of the tendon diameter, including complete tear) [14]. In this study, grade I and grade II lesions were defined as LHBT tears (Fig. 2). Among the 582 cases, LHBT tears were observed in 46.4% (270/582); 53.6% (312/582) were grade 0; 32.3% (188/582) were grade I; and 14.1% (82/582) were grade II. Among the grade II tears, 9.8% (8/82) involved complete biceps tendon tears. Of the LHBT tears, 38.1% (103/270) involved subluxation, and 7.0% (19/270) involved dislocation. Table 1 summarizes the prevalence of LHBT tear associated with each variable related to RCT.

### Statistical analysis

The strengths of associations between LHBT tear and various studied variables were determined by calculating the odds ratios (ORs), with 95% confidence intervals (CIs), using logistic regression analyses. Univariate logistic regression analyses were performed for all variables; forward stepwise multivariable logistic regression analysis was then performed on variables demonstrating significant associations after assessments of multicollinearity. Multicollinearity was considered absent when both the variance inflation factor (VIF) and condition index were < 10 among the variables [36]. The Hosmer-Lemeshow, Stukel, and Osius-Rojek tests were used to determine the goodness of fit for the multivariable logistic regression model. Finally, the model with the lowest value of Akaike information criterion was selected from among the combinations of associated factors included in the multivariable analysis [37]. The number of variables to be included in the final model was limited to 15 events per variable to avoid the problem of overfitting [38].

All statistical analyses were performed using the SPSS software program (IBM SPSS Statistics for Windows, Version 24.0. Armonk, NY: IBM Corp.), except the Stukel and the Osjus-Rojek tests, which were analyzed using the rms software package in R (http://www.r-project.org). Significance of the logistic analyses was set at *p* < .05. Significance of the Hosmer-Lemeshow, the Stukel, and the Osjus-Rojek tests were set at *p* > .05.

To determine the diagnostic values of the combinations of the significant factors in the multivariable logistic analysis, we calculated pre- and post-test probabilities, pre- and post-test odds, and positive and negative likelihood ratios, using the two-by-two table method. In cases involving continuous or ordinal values, receiver operating characteristic curves were used to detect the cufoff value of each significant variable. Likelihood ratios indicate the values of various clinical tests for increasing the certainty of a diagnosis. In the case of a single test, a likelihood ratio of > 10 is sufficient to rule in the target condition; there, a likelihood ratio of < 0.1 is sufficient to rule out the target condition [39].

## Results

In the univariate analyses, age, factors related to RCT, hs-CRP > 1 mg/L, and pain VAS were significant variables. The factors related to RCT were the presence, thickness, size of RCT(supraspinatus, infraspinatus, and subscapularis tendon tear), retraction degree of Patte, Goutallier’s grade, GFDI, the tangent sign, and the occupation ratio. The ORs for all variables with 95% CIs are presented in Table 2.

**Table 2:** Strengths of associations between long head of biceps tendon tears and specific factors related to rotator cuff tears in the univariate analyses

Variables	Odds ratio (95% confidence interval)	*p*-value
Age*	1.6 (1.4–1.9)	< 0.01
Male gender	1.3 (0.9–1.9)	0.08
BMI	0.9 (0.9–1.1)	0.64
Smoking	1.1 (0.8–1.6)	0.49
Dominant side-involvement	1.1 (0.8–1.6)	0.47
Diabetes	1.3 (0.8–2.0)	0.33
Hypertension	1.0 (0.7–1.4)	0.89
Hyperthyroidism	1.2 (0.6–2.4)	0.68
Hypothyroidism	2.7 (0.8–8.7)	0.11
ASA grade	1.2 (0.9–1.5)	0.23
Prevalence of dyslipidemia	0.8 (0.4–1.6)	0.44
Hyper-cholesterolemia	0.9 (0.6–1.3)	0.63
Hyper-LDLemia	0.8 (0.6–1.1)	0.19
Hyper-TGmia	0.9 (0.6–1.3)	0.42
Hypo-HDLemia	0.9 (0.6–1.2)	0.45
Hyper-non-HDLemia	0.9 (0.6–1.2)	0.40
Rotator cuff tears*	1.9 (1.3–2.6)	< 0.01
Supraspinatus tendon tear*	1.6 (1.3–2.0)	< 0.01
Infraspinatus tendon tear*	1.8 (1.5–2.3)	< 0.01
Subscapularis tendon tear*	2.4 (1.8–3.1)	< 0.01
Depth of rotator cuff tears*	1.6 (1.3–2.0)	< 0.01
Size of rotator cuff tear*	1.4 (1.2–1.5)	< 0.01
Retraction degree of Patte*	2.5 (2.0–2.9)	< 0.01
Goutallier’s fatty degeneration grade*	1.6 (1.3–1.9)	< 0.01
Global fatty degeneration index*	1.9 (1.5–2.4)	< 0.01
Positive tangent sign*	1.7 (1.5–2.1)	< 0.01
Occupation ratio*	1.8 (1.4–2.4)	< 0.01
hs-CRP > 1(mg/L)*	2.3 (1.8–2.8)	< 0.01
Pain VAS*	1.6 (1.4–1.9)	< 0.01

In the multivariable analysis, age (OR, 1.5 [95% CI, 1.3 to 1.8]; *p* < .01), subscapularis tendon tear (OR, 1.6 [95% CI, 1.2 to 2.2]; *p* < .01), retraction degree of Patte (OR, 2.0 [1.6 to 2.4]; *p* < .01), hs-CRP > 1 mg/L (OR, 1.6 [95% CI, 1.2 to 2.0]; *p* < .01), and pain VAS (OR, 1.3 [95% CI, 1.1 to 1.5]; *p* < .01) were significantly associated with LHBT tear. The VIF, condition index, and ORs with 95% CIs for all of the variables are summarized in Table 3. The *p-*values of the Hosmer-Lemeshow, Stukel, and Osjus-Rojek tests were 0.41, 0.39, and 0.21, respectively, indicating a good fit for each.

**Table 3:** Strengths of associations between long head of biceps tendon tears and specific factors related to rotator cuff tears in the multivariable analysis

Variables	Odds ratio (95% confidence interval)	*p*-value
Age	1.5 (1.3–1.8)	< 0.01
Subscapularis tendon tear	1.6 (1.2–2.2)	< 0.01
Retraction degree of Patte	2.0 (1.6–2.4)	< 0.01
hs-CRP > 1(mg/L)	1.6 (1.2–2.0)	< 0.01
Pain VAS	1.3 (1.1–1.5)	< 0.01

Variance inflation factor = 1.33 and condition index = 3.66. Akaike information criterion and area under receiver operator characteristics curve were 578, and 0.8

The cutoff value and the area under curve for age were 66.5 years and 0.63 ([95% CI, 0.59 to 0.68]; *p* <  0.001). The cutoff value and the area under curve for pain VAS were 6.5 and 0.66 ([95% CI, 0.62 to 0.71]; *p* <  0.001). The cutoff value and the area under curve for degree of Patte were 1.5 and 0.72 ([95% CI, 0.67 to 0.76]; *p* <  0.001).

The diagnostic accuracy of the combination of the five variables that were higher than their cutoff values (age ≥ 67 years, subscapularis tendon tear, grade of Patte ≥2, hs-CRP > 1 mg/L, and pain VAS ≥ 7) yielded a strong positive likelihood ratio of 19.07 and a high post-test probability of 0.96 (96%). The results of the probabilities, odds, and likelihood ratios of each variable and of the combinations of variables are summarized in Table 4.

**Table 4:** The likelihood ratios and post-test probabilities for combining clinical variables according to multivariable logistic regression analysis results

	No. (%) of Patients with Positive Test Results		Pretest Probability	Pretest Odds	Post-Test Probability	Post-Test Odds	Positive likelihood Ratio	Negative likelihood Ratio
	Biceps tendon tear group	Biceps tendon intact group						
Positive for five variables	12.22% (33/270)	0.64% (2/312)	0.46	0.87	0.96	16.50	19.07	0.88
Positive for four variables	13.70% (37/270)	1.60% (5/312)	0.46	0.87	0.88	7.41	8.55	0.88
Positive for three variables	27.04% (73/270)	3.31% (10/302)	0.46	0.87	0.87	7.33	8.44	0.75
Positive for two variables	30.00% (81/270)	6.73% (21/312)	0.46	0.87	0.79	3.86	4.46	0.75
Positive for age ≥ 67	51.11% (138/270)	24.68% (77/312)	0.46	0.87	0.64	1.79	2.07	0.65
Positive for subscapularis tendon tear	54.44% (147/270)	23.72% (74/312)	0.46	0.87	0.68	2.01	2.30	0.60
Positive for degree of Patte ≥2	54.44% (147/270)	13.78% (43/312)	0.46	0.87	0.71	2.71	3.95	0.53
Positive for hs-CRP > 1 mg/L	60.74% (164/270)	23.72% (74/312)	0.46	0.87	0.61	1.50	1.92	0.71
Positive for pain VAS ≥7	59.63% (161/270)	27.24% (85/312)	0.46	0.87	0.65	1.89	2.19	0.55

A total of 582 patients (270 in the biceps tendon tear group and 312 in the biceps tendon intact group) were included in this analysis. The cutoff values of age ≥ 67 years, degree of Patte ≥2, and pain VAS ≥7 were determined using the ROC curve method. The combination of four positive variables did not include the variable of hs-CRP > 1 mg/L. The results of the combinations of triads and of pairs of positive variables were derived from the values of the highest positive likelihood ratios among those triads and pairs

## Discussion

This study found that age, subscapularis tendon tear, retraction degree of Patte, hs-CRP > 1 mg/L, and pain VAS are significantly associated with LHBT tear.

Age was a significantly associated factor with LHBT tear in this study. Previously, Carter et al. [40] reported that ruptures of the LHBT are most common in people over 50 years of age. Minagawa et al. [41] demonstrated the increasing prevalence of LHBT tear with the advancement in patients’ ages: 0% with LHBT tears in their 40s or younger, 10.7% in their 50s, 15.2% in their 60s, 26.5% in their 70s, and 36.6% in their 80s or older. Yamamoto et al. [42] reported that the rate of LHBT tear increased with age. Refior and Sowa [43], reporting their histologic study, stated that age was significantly related to LHBT tendon degeneration. Our findings of a close association between age and LHBT tear confirm the results of those previous studies.

Many authors have suggested that subscapularis tendon tears have a significant association with LHBT lesions [14, 44–46]. Chen et al. [4] reported that 97% of RCT with subscapularis tendon involvement had LHBT lesions and that multiple tendon tears suggested advanced LHBT lesions. Lafosse et al. [14] suggested that medial instability of LHBT was associated with subscapularis tendon tear and that posterolateral instability was associated with supraspinatus tendon tear. Biomechanical studies have demonstrated that the increase in load of LHBT is greater after subscapularis tendon tear than after infraspinatus tendon tears [47, 48]. Our study confirmed those previous studies’ finding that subscapularis tendon tear is significantly associated with LHBT tear.

RCT is a well-known risk factor for LHBT tear [11–13]. The chronicity of RCT has been defined as the duration of a tear and muscle atrophy; the fatty infiltration of the rotator cuff muscle is a characteristic change that represents the chronicity of the tear [49, 50]. Various methods have been developed to grade muscle atrophy and fatty infiltration, including the Goutallier’s grade, GFDI, tangent sign, and occupation ratio [30–34]. The retraction degree of Patte does not directly reflect muscle atrophy or fatty degeneration. However, according to the Thomazeau et al. [51] study, the atrophy of the supraspinatus muscle and the retraction grade of the rotator cuff tendon are highly correlated. Chen et al. [4] have reported that chronic RCT with symptom duration longer than 3 months or with massive size tear is associated with LHBT lesions. According to our univariate study, all indexes regarding chronicity of RCT are associated with LHBT tear. This finding strongly suggests that chronicity of RCT is a risk factor for LHBT tear. Wu et al. [13] also indicated that the coexisting RCT’s size plays a role in the severity of tendinopathy of LHBT. Those previous studies support the retraction degree of Patte as an independent risk factor for LHBT tear.

Serum hs-CRP, as a marker of inflammation, has been developed to increase sensitivity in the detection of lower levels of CRP, < 1 mg/L [23, 52]. Elevated levels of hs-CRP have been reported to be associated with chronic inflammatory diseases, including osteoarthritis, chronic lower back pain, sciatic pain, and frozen shoulder [26, 27, 29]. Carp et al. [25] suggested that the severity of upper-extremity overuse disorders is more correlated with an increase in CRP than with an increase in other inflammatory biomarkers. Kowalczuk et al. [11] reported that inflammation in the rotator cuff interval is signaled by LHBT tear, which is a harbinger of rotator cuff disease. Murthi et al. [5] histologically demonstrated that chronic inflammatory change is present in macroscopically degenerated or fibrillated LHBT. Zabrzynski et al. [53] also histologically demonstrated the presence of the marginal inflammation process in LHBT with tendinopathy. Carp et al. [25] suggested that elevated CRP can be initiated by a local response and is proportionally amplified in the presence of greater tissue injury and inflammation. Inflammation is known to be associated with the sensitization of sensory neurons composing the pain pathway that promotes pain sensations [54]. These previous studies support the current study’s finding of a significant association between hs-CRP and LHBT tear.

This study suggests this as the best combination of determinations for diagnosing LHBT tear: age ≥ 67 years, subscapularis tendon tear, grade of Patte ≥2, hs-CRP > 1, and pain VAS ≥ 7, which yielded a strong positive likelihood ratio of 19.07. The combination of four tests, excluding the factor of hs-CRP > 1 mg/L, yielded a moderate likelihood ratio of 8.55 (Table 4). These findings suggest that hs-CRP > 1 mg/L increases diagnostic accuracy for LHBT tear. Positive findings for all five of the variables would allow one to rule in LHBT tear [55]. However, negative findings for all five of the variables would not allow one to rule out LHBT tear because the negative likelihood ratio was 0.88, which is higher than 0.1 [55].

The present study has several limitations. First, although we evaluated the biceps tendons after intra-articular retraction, we did not completely evaluate hidden biceps lesions, which are those located at a point distal from the transverse humeral ligament. Second, the current study did not evaluate the severity of LHBT tear based on histological findings. Third, this study did not evaluate hypertrophied biceps tendons, which are known to be closely related to RCTs. In the current study, we found 16 cases of hypertrophied biceps tendon without tear. Those hypertrophied biceps tendons were all in conjunction with large or massive chronic rotator cuff tears. However, we did not include those cases in the category of LHBT tear. Finally, given the design of a cross-sectional study, there are limitations in exactly specifying whether the origin of the inflammation is the biceps tendon, the rotator interval, or accompanying chronic inflammatory diseases.

## Conclusions

Serum hs-CRP > 1 mg/L is an independent risk factor for LHBT tear, along with the expected risk factors of age, subscapularis tendon tear, retraction degree of Patte, and pain VAS. Serum hs-CRP > 1 mg/L increases the diagnostic accuracy for LHBT tear.

## Data Availability

The dataset generated and/or analyzed during the current study are not publicly available since we did not obtained the right to data publicity even in de-identified form from the start of the study.

## Abbreviations

ASA: American Society of Anesthesiologists
BMI: Body mass index
CI: Confidence interval
CRP: C-reactive protein
GFDI: Global fatty degeneration index
HS-CRP: High-sensitivity C-reactive protein
LHBT: Long head of biceps tendon
OR: Odds ratio
RCT: Rotator cuff tear
VAS: Visual analog scale
VIF: Variance inflation factor

## References

[1] Raney EB, Thankam FG, Dilisio MF, Agrawal DK (2017). Pain and the pathogenesis of biceps tendinopathy. Am J Transl Res.

[2] Ahrens PM, Boileau P (2007). The long head of biceps and associated tendinopathy. J Bone Joint Surg Br.

[3] Gill HS, El Rassi G, Bahk MS, Castillo RC, McFarland EG (2007). Physical examination for partial tears of the biceps tendon. Am J Sports Med.

[4] Chen CH, Hsu KY, Chen WJ, Shih CH (2005). Incidence and severity of biceps long head tendon lesion in patients with complete rotator cuff tears. J Trauma.

[5] Murthi AM, Vosburgh CL, Neviaser TJ (2000). The incidence of pathologic changes of the long head of the biceps tendon. J Shoulder Elb Surg.

[6] Patton WC, McCluskey GM (2001). Biceps tendinitis and subluxation. Clin Sports Med.

[7] Borrero CG, Costello J, Bertolet M, Vyas D (2018). Effect of patient age on accuracy of primary MRI signs of long head of biceps tearing and instability in the shoulder: an MRI-arthroscopy correlation study. Skelet Radiol.

[8] Urita A, Funakoshi T, Amano T, Matsui Y, Kawamura D, Kameda Y (2016). Predictive factors of long head of the biceps tendon disorders-the bicipital groove morphology and subscapularis tendon tear. J Shoulder Elb Surg.

[9] Braun S, Horan MP, Elser F, Millett PJ (2011). Lesions of the biceps pulley. Am J Sports Med.

[10] Leite MJ, Sa MC, Lopes MJ, Matos RM, Sousa AN, Torres JM (2019). Coracohumeral distance and coracoid overlap as predictors of subscapularis and long head of the biceps injuries. J Shoulder Elb Surg.

[11] Kowalczuk M, Kohut K, Sabzevari S, Naendrup JH, Lin A (2018). Proximal long head biceps rupture: a predictor of rotator cuff pathology. Arthroscopy.

[12] Beall DP, Williamson EE, Ly JQ, Adkins MC, Emery RL, Jones TP (2003). Association of biceps tendon tears with rotator cuff abnormalities: degree of correlation with tears of the anterior and superior portions of the rotator cuff. AJR AJR Am J Roentgenol.

[13] Wu PT, Jou IM, Yang CC, Lin CJ, Yang CY, Su FC (2014). The severity of the long head biceps tendinopathy in patients with chronic rotator cuff tears: macroscopic versus microscopic results. J Shoulder Elb Surg.

[14] Lafosse L, Reiland Y, Baier GP, Toussaint B, Jost B (2007). Anterior and posterior instability of the long head of the biceps tendon in rotator cuff tears: a new classification based on arthroscopic observations. Arthroscopy.

[15] Cofield RH, Parvizi J, Hoffmeyer PJ, Lanzer WL, Ilstrup DM, Rowland CM (2001). Surgical repair of chronic rotator cuff tears. A prospective long-term study. J Bone Joint Surg Am.

[16] Tosounidis T, Hadjileontis C, Triantafyllou C, Sidiropoulou V, Kafanas A, Kontakis G (2013). Evidence of sympathetic innervation and alpha1-adrenergic receptors of the long head of the biceps brachii tendon. J Orthop Sci.

[17] Alpantaki K, McLaughlin D, Karagogeos D, Hadjipavlou A, Kontakis G (2005). Sympathetic and sensory neural elements in the tendon of the long head of the biceps. J Bone Joint Surg Am.

[18] Boileau P, Baque F, Valerio L, Ahrens P, Chuinard C, Trojani C (2007). Isolated arthroscopic biceps tenotomy or tenodesis improves symptoms in patients with massive irreparable rotator cuff tears. J Bone Joint Surg Am.

[19] Walch G, Edwards TB, Boulahia A, Nove-Josserand L, Neyton L, Szabo I (2005). Arthroscopic tenotomy of the long head of the biceps in the treatment of rotator cuff tears: clinical and radiographic results of 307 cases. J Shoulder Elb Surg.

[20] Hsu SH, Miller SL, Curtis AS (2008). Long head of biceps tendon pathology: management alternatives. Clin Sports Med.

[21] Schmalzl J, Plumhoff P, Gilbert F, Gohlke F, Konrads C, Brunner U, Jakob F, Ebert R, Steinert AF (2019). The inflamed biceps tendon as a pain generator in the shoulder: A histological and biomolecular analysis. J Orthop Surg (Hong Kong).

[22] Oh SW, Moon JD, Park SY, Jang HJ, Kim JH, Nahm KB (2005). Evaluation of fluorescence hs-CRP immunoassay for point-of-care testing. Clin Chem.

[23] Rifai N, Ridker PM (2001). High-sensitivity C-reactive protein: a novel and promising marker of coronary heart disease. Clin Chem.

[24] Pietzner M, Kaul A, Henning AK, Kastenmuller G, Artati A (2017). Comprehensive metabolic profiling of chronic low-grade inflammation among generally healthy individuals. BMC Med.

[25] Carp SJ, Barbe MF, Winter KA, Amin M, Barr AE (2007). Inflammatory biomarkers increase with severity of upper-extremity overuse disorders. Clin Sci (Lond).

[26] Bulgen DY, Binder A, Hazleman BL, Park JR (1982). Immunological studies in frozen shoulder. J Rheumatol.

[27] Spector TD, Hart DJ, Nandra D, Doyle DV, Mackillop N, Gallimore JR (1997). Low-level increases in serum C-reactive protein are present in early osteoarthritis of the knee and predict progressive disease. Arthritis Rheum.

[28] Punzi L, Ramonda R, Oliviero F, Sfriso P, Mussap M, Plebani M (2005). Value of C reactive protein in the assessment of erosive osteoarthritis of the hand. Ann Rheum Dis.

[29] Sturmer T, Raum E, Buchner M, Gebhardt K, Schiltenwolf M, Richter W (2005). Pain and high sensitivity C reactive protein in patients with chronic low back pain and acute sciatic pain. Ann Rheum Dis.

[30] Patte D. Classification of rotator cuff lesions. Clin Orthop Relat Res. 1990;254:81-6. 2323151

[31] Goutallier D, Postel JM, Bernageau J, Lavau L, Voisin MC. Fatty muscle degeneration in cuff ruptures. Pre- and postoperative evaluation by CT scan. Clin Orthop Relat Res. 1994;304:78-83. 8020238

[32] Goutallier D, Postel JM, Gleyze P, Leguilloux P, Van Driessche S (2003). Influence of cuff muscle fatty degeneration on anatomic and functional outcomes after simple suture of full-thickness tears. J Shoulder Elb Surg.

[33] Zanetti M, Gerber C, Hodler J (1998). Quantitative assessment of the muscles of the rotator cuff with magnetic resonance imaging. Investig Radiol.

[34] Thomazeau H, Rolland Y, Lucas C, Duval JM, Langlais F (1996). Atrophy of the supraspinatus belly. Assessment by MRI in 55 patients with rotator cuff pathology. Acta Orthop.

[35] Cofield RH (1982). Subscapular muscle transposition for repair of chronic rotator cuff tears. Surg Gynecol Obstet.

[36] Belsley DA (2004). Regression diagnostics: identifying influential data and sources of collinearity.

[37] Bozdogan H (1987). Model selection and Akaike’s information criterion (AIC): the general theory and its analytical extensions. Psychometrika.

[38] Peduzzi P, Concato J, Kemper E, Holford TR, Feinstein AR (1996). A simulation study of the number of events per variable in logistic regression analysis. J Clin Epidemiol.

[39] GG JR, Lijmer J (2002). Diagnostic tests.

[40] Carter AN, Erickson SM (1999). Proximal biceps tendon rupture: primarily an injury of middle age. Phys Sportsmed.

[41] Minagawa H, Itoi E (2006). Clinical relevance of the rotator cuff in the shoulder with pain and dysfunction [in Japanese]. Kansetsugeka.

[42] Yamamoto A, Takagishi K, Osawa T, Yanagawa T, Nakajima D, Shitara H (2010). Prevalence and risk factors of a rotator cuff tear in the general population. J Shoulder Elb Surg.

[43] Refior HJ, Sowa D (1995). Long tendon of the biceps brachii: sites of predilection for degenerative lesions. J Shoulder Elb Surg.

[44] Li X, Fallon J, Egge N, Curry EJ, Patel K, Owens BD (2013). MRI study of associated shoulder pathology in patients with full-thickness subscapularis tendon tears. Orthopedics..

[45] Bennett WF (2001). Subscapularis, medial, and lateral head coracohumeral ligament insertion anatomy. Arthroscopic appearance and incidence of "hidden" rotator interval lesions. Arthroscopy.

[46] Gleason PD, Beall DP, Sanders TG, Bond JL, Ly JQ, Holland LL (2006). The transverse humeral ligament: a separate anatomical structure or a continuation of the osseous attachment of the rotator cuff?. Am J Sports Med.

[47] Su WR, Budoff JE, Luo ZP (2009). The effect of anterosuperior rotator cuff tears on glenohumeral translation. Arthroscopy..

[48] Su WR, Budoff JE, Luo ZP (2010). The effect of posterosuperior rotator cuff tears and biceps loading on glenohumeral translation. Arthroscopy..

[49] Melis B, Wall B, Walch G (2010). Natural history of infraspinatus fatty infiltration in rotator cuff tears. J Shoulder Elb Surg.

[50] Goutallier D, Le Guilloux P, Postel JM, Radier C, Bernageau J, Zilber S (2011). Acromio humeral distance less than six millimeter: its meaning in full-thickness rotator cuff tear. Orthop Traumatol Surg Res.

[51] Thomazeau H, Boukobza E, Morcet N, Chaperon J, Langlais F (1997). Prediction of rotator cuff repair results by magnetic resonance imaging. Clin Orthop Relat Res.

[52] Ridker PM, Cook N (2004). Clinical usefulness of very high and very low levels of C-reactive protein across the full range of Framingham risk scores. Circulation.

[53] Zabrzynski J, Paczesny L, Lapaj L, Grzanka D, Szukalski J (2017). Is the inflammation process absolutely absent in tendinopathy of the long head of the biceps tendon? Histopathologic study of the long head of the biceps tendon after arthroscopic treatment. Pol J Pathol.

[54] Inglis JJ, Nissim A, Lees DM, Hunt SP, Chernajovsky Y, Kidd BL (2005). The differential contribution of tumour necrosis factor to thermal and mechanical hyperalgesia during chronic inflammation. Arthritis Res Ther.

[55] Walton J, Mahajan S, Paxinos A, Marshall J, Bryant C, Shnier R (2004). Diagnostic values of tests for acromioclavicular joint pain. J Bone Joint Surg Am.

